# Integrating exome sequencing into a diagnostic pathway for epileptic encephalopathy: Evidence of clinical utility and cost effectiveness

**DOI:** 10.1002/mgg3.355

**Published:** 2018-01-04

**Authors:** Elizabeth E. Palmer, Deborah Schofield, Rupendra Shrestha, Tejaswi Kandula, Rebecca Macintosh, John A. Lawson, Ian Andrews, Hugo Sampaio, Alexandra M. Johnson, Michelle A. Farrar, Michael Cardamone, David Mowat, George Elakis, William Lo, Ying Zhu, Kevin Ying, Paula Morris, Jiang Tao, Kerith‐Rae Dias, Michael Buckley, Marcel E. Dinger, Mark J. Cowley, Tony Roscioli, Edwin P. Kirk, Ann Bye, Rani K. Sachdev

**Affiliations:** ^1^ Sydney Children's Hospital Randwick NSW Australia; ^2^ School of Women's and Children's Health UNSW Medicine The University of New South Wales Sydney NSW Australia; ^3^ Genetics of Learning Disability Service Waratah NSW Australia; ^4^ The Garvan Institute for Medical Research Darlinghurst, Sydney NSW Australia; ^5^ Faculty of Pharmacy The University of Sydney Sydney NSW Australia; ^6^ The Murdoch Children's Research Institute Melbourne Vic. Australia; ^7^ SEALS pathology Randwick NSW Australia; ^8^ St Vincent's Clinical School University of New South Wales Sydney NSW Australia

**Keywords:** diagnosis, epilepsy, genomics, health economics

## Abstract

**Background:**

Epileptic encephalopathies are a devastating group of neurological conditions in which etiological diagnosis can alter management and clinical outcome. Exome sequencing and gene panel testing can improve diagnostic yield but there is no cost‐effectiveness analysis of their use or consensus on how to best integrate these tests into clinical diagnostic pathways.

**Methods:**

We conducted a retrospective cost‐effectiveness study comparing trio exome sequencing with a standard diagnostic approach, for a well‐phenotyped cohort of 32 patients with epileptic encephalopathy, who remained undiagnosed after “first‐tier” testing. Sensitivity analysis was included with a range of commercial exome and multigene panels.

**Results:**

The diagnostic yield was higher for the exome sequencing (16/32; 50%) than the standard arm (2/32; 6.2%). The trio exome sequencing pathway was cost‐effective compared to the standard diagnostic pathway with a cost saving of AU$5,236 (95% confidence intervals $2,482; $9,784) per additional diagnosis; the standard pathway cost approximately 10 times more per diagnosis. Sensitivity analysis demonstrated that the majority of commercial exome sequencing and multigene panels studied were also cost‐effective. The clinical utility of all diagnoses was reported.

**Conclusion:**

Our study supports the integration of exome sequencing and gene panel testing into the diagnostic pathway for epileptic encephalopathy, both in terms of cost effectiveness and clinical utility. We propose a diagnostic pathway that integrates initial rapid screening for treatable causes and comprehensive genomic screening. This study has important implications for health policy and public funding for epileptic encephalopathy and other neurological conditions.

## 1 INTRODUCTION

Infantile‐onset epileptic encephalopathy (EE) is a descriptive term for a group of severe epilepsy disorders characterized by early onset of drug‐resistant seizures, with developmental stagnation or regression (Berg et al., 2010). The estimated incidence of EE is 4.3 per 10,000 live births per year (Hino‐Fukuyo et al., 2009). EE results in a high burden of care for families and the health service (Beghi et al., 2005), a significant risk of comorbidity, and a shortened life span (Khan & Al Baradie, 2012). Diagnosis is challenging as etiologies are numerous with significant phenotypic overlap; these include nongenetic causes as well as over 200 monogenic seizure disorders, inborn errors of metabolism, and intellectual disability syndromes (McTague et al., 2016). The benefits of a timely etiological diagnosis are clear (Reif et al., 2017): allowing personalized management guiding choice of antiepileptic (Consortium E, 2015) or metabolic treatment (van Karnebeek et al., 2014), health surveillance for recognized comorbidities, accurate estimation of chance of recurrence in the family, reproductive options counseling, provision of “closure,” and access to specific support groups for families (Berkovic, 2015).

A typical diagnostic approach for EE encompasses two steps: first‐ and second‐tier assessments. First‐tier assessment includes neurological and clinical genetics review, screening for metabolic disorders, neuroimaging, neurophysiology, chromosomal microarray, and targeted genetic testing for clinically suspected conditions (Kamien et al., 2012; Mercimek‐Mahmutoglu et al., 2015). This allows for diagnosis of EE secondary to cortical malformations/hemorrhage or strokes, perinatal hypoxia–ischemia, focal lesions amenable to surgery, some metabolic and mitochondrial disorders, clinically recognizable syndromes such as tuberous sclerosis, pathogenic chromosomal copy number variants, and electroclinical syndromes associated with a particular gene such as *SCN1A*‐related Dravet syndrome (MIM 607208). However, 40%–50% of patients with EE remain undiagnosed after first‐tier assessment (Osborne et al., 2010; Poulat et al., 2014). Second‐tier assessment involves repeated clinical assessments, further neuroimaging and metabolic studies, and sequencing of individual genes (Kamien et al., 2012; Mercimek‐Mahmutoglu et al., 2015). This approach often represents a diagnostic odyssey for patients, their families, and clinicians: invasive, costly, time‐consuming, and with low diagnostic yield (<10%) (Mercimek‐Mahmutoglu et al., 2015).

The low diagnostic yield of standard second‐tier testing reflects the difficulty in targeting genetic testing for EE: it is increasingly recognized that genes rarely respect the concept of electroclinical syndromes, such as those proposed by the International League Against Epilepsy ILAE (Berg et al., 2010). Variants in one gene may cause different electroclinical syndromes, and conversely each electroclinical syndrome has an ever‐growing range of genetic causes (Gursoy & Ercal, 2016; Mastrangelo, 2015; McTague et al., 2016; Palmer et al., 2017). In addition, the field is rapidly changing. OMIM currently lists 56 genetic causes of infantile onset EE (source: Online Mendelian Inheritance in Man website accessed June 2017, http://www.omim.org/phenotypicSeries/PS308350), but there are well over 200 monogenetic conditions in which EE has been reported (Gursoy & Ercal, 2016). However, there is now a substantial body of work demonstrating that application of high‐throughput sequencing (HTS) via targeted multigene panels or exome sequencing (ES) improves diagnostic yield in EE by 20%–40% (Chambers, Jansen, & Dhamija, 2016; Consortium E‐R, Project EPG, Consortium EK, 2014; Helbig et al., 2016; Kwong et al., 2015; Lemke et al., 2012; Trump et al., 2016); HTS has the potential to minimize this invasive and expensive “diagnostic odyssey.” Most reports claim that this implies cost effectiveness (Helbig et al., 2016; Lemke et al., 2012), but solid health economic data are lacking.

The closest analysis to a cost‐effectiveness study in an EE cohort was undertaken by Joshi et al. (2016). The authors reported the cost of first‐ and second‐tier testing for four patients from three families. This study reported second‐tier standard testing (excluding consultation costs) for EE of between US$9,015 and US$35,480. However, all of the patients in Joshi et al.'s study had a genetic diagnosis only after exome sequencing, and no patients who may have had a diagnosis using the standard diagnostic pathway were included. While this study produces costs of different testing methods for a small sample of patients, cost effectiveness of ES over a standard diagnostic path cannot be inferred as the study only reports on diagnosed patients, and only those diagnosed by ES. For cost‐effectiveness analysis, data are required from a cohort that encompasses *all* patients who incurred costs for exome sequencing, including patients who did not reach a diagnosis as well those who received a diagnosis, from both ES *and* the comparative standard diagnostic approach.

It is critical to provide timely data about the health costs of genomic testing in conditions such as EE (Cartwright, 1997) to inform policymakers. However, a prospective cost‐effectiveness study with randomization to standard versus ES diagnostic pathway would be unethical, given the well‐established clinical benefits of establishing a molecular diagnosis and the improved diagnostic yield with ES/multigene panel over standard testing in EE (Helbig et al., 2016). We thus aimed to supply a cost‐effectiveness analysis of a trio ES approach compared with standard testing, in a well‐defined clinical cohort of 32 patients with EE who were undiagnosed after first‐tier testing, using a well‐established counterfactual health economics study design. With this study design, patients are used as their own controls and cost‐effective comparison is done as if ES had been done early in the diagnostic pathway. The deliberate focus of the study was on patients undiagnosed after first‐tier testing, as the most pressing clinical need is to develop an optimal diagnostic pathway for this group of patients. The study was performed in a pediatric children's hospital and, to our knowledge, is the first to provide a robust cost‐effectiveness study of ES in EE.

## 2 SUBJECTS/MATERIALS AND METHODS

### 2.1 Ethical compliance

The research was approved by the ethics committee from The Sydney Children's Hospital Network and the Prince of Wales Hospital Campus, Sydney, Australia (HREC ref no. 13/094).

### 2.2 Study design/Patient cohort and inclusion/exclusion criteria

**Figure 1:**
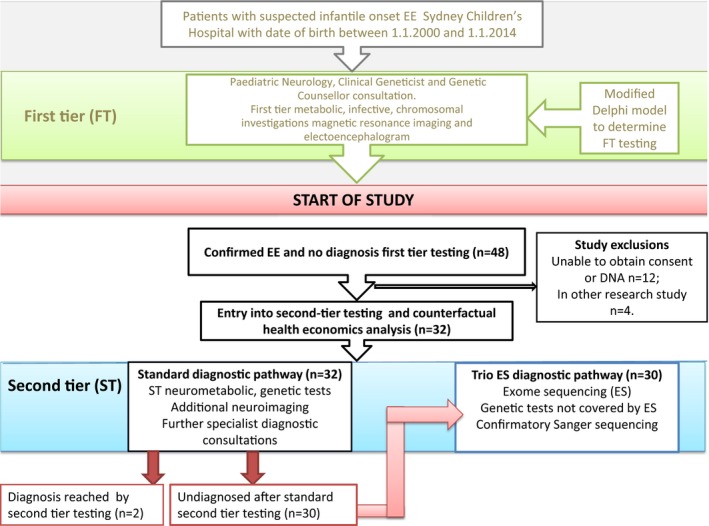
Flowchart of study design. EE, epileptic encephalopathy; ES, exome sequencing; DNA, deoxyribonucleic acid

An overview of the study design is provided in Figure 1. There were 48 infantile onset EE patients of Sydney Children's Hospital Australia, born between 1 January 2000 and 31 December 2013, who remained undiagnosed after first‐tier assessment and were eligible for enrollment in the study. All patients were required to fulfill strict diagnostic criteria for infantile‐onset EE based on the current ILAE definition (Berg et al., 2010): namely they were required to have (a) drug‐resistant epilepsy for a minimum of 6 months, (b) seizure onset accompanied by adverse impact on development such as developmental stagnation or regression, (c) an infantile‐onset of seizures (before 18 months), and (d) at least one electroencephalogram (EEG) that was significantly abnormal with diffusely poorly organized background and marked bihemispheric epileptogenic activity (e.g., hypsarrhythmia, multifocal). Proband and parental DNA needed to be available. Patients were excluded if they had a clear genetic/other etiological diagnosis previously established on first‐tier assessment, for example, tuberous sclerosis (MIM: 605284), *SCN1A*‐related Dravet syndrome (MIM 182389), major structural/focal anomaly on neuroimaging, vascular stroke, head injury, infection, ischemia, if the primary neurologist or clinical geneticist were not in agreement with the enrollment of family in study, or if the patient were already entered into another research genetic study.

First‐tier assessment comprised investigations/consultations considered appropriate for initial investigation of a patient with EE regardless of the availability of exome sequencing (ES), as the results of these could potentially alter immediate clinical management, and establish a number of important, and potentially treatable, diagnoses. Consensus regarding first‐tier assessment was reached by consultation with all clinical neurologists of the Paediatric Neurology Department of SCH using a modified Delphi approach (Powell, 2003), and was consistent with current literature recommendations (Kamien et al., 2012; Mercimek‐Mahmutoglu et al., 2015). Assessments and tests considered as “first tier” in this study included pediatric neurology and clinical geneticist consultations, a consultation with a genetic counselor including the consent process for genetic testing, brain magnetic resonance imaging (MRI), a routine 20 min EEG, “first‐tier” urine, blood, and cerebrospinal fluid studies, chromosomal microarray, and single‐gene testing if a single monogenic condition was specifically indicated by the patient's phenotype (e.g., *SCN1A* testing if the patient fulfilled diagnostic criteria for Dravet syndrome). Full details of the first‐tier tests and consultation, with associated costs, are included in Table S1.

Twelve patients were excluded as informed consent or sufficient DNA was unable to be obtained and a further four were excluded as they were enrolled in other research studies. A cohort of 32 patients remained. Table S2 summarizes the demographic characteristics of the cohort and phenotypic details of the cohort are provided in Table 1 and in more detail in Table S3. The average age of undiagnosed patients entering our study was 46.6 months.

**Table 1:** Summary of genetic results from second‐tier testing

Fam	Age (years) Gender Parental consanguinity?	Phenotype (seizure onset and type(s); EEG semiology; developmental and neurological features)	Method Diagnosis	ACMG Classification Previously reported (PMID)	Variant (s) GRCh37	Clinical utility of result
1	11 female NC.	Seizure onset 4 mo: IS + MF/tonic EEG: MFE +AB. Profound DD, dystonia, cortical inattention, slowly deteriorating course.	Trio ES	LP Yes (PMID: 10888601)	NM_000026.2 (*ADSL*): c.[1288G>A]; [1370T>C]	Palliative care initiated. No further intensive care admissions. Family closure. End diagnostic odyssey.
2	11 male NC	Seizure onset 4 mo: Tonic, CP, GTC + focal EEG: MFE + AB Profound DD, regression, hypotonia, progressive postnatal microcephaly and cerebral atrophy.	Trio ES	P Yes (PMID: 26318253)	NM_183356.3 (*ASNS*): c.[866G>C]; [1010C>T]	Reduced invasive/costly diagnostic investigations. Family closure. End diagnostic odyssey.
3	1 female C (deceased)	Seizure onset 4 mo: focal to intractable MF. EEG: modified hyps, MFE + AB Profound DD, regression, congenital retinopathy.	Trio ES	LP Yes (PMID: 27270415)	NM_022786.1 (*ARV1*): c.[294 + 1G>A]; c.[294 + 1G>A]	Reproductive planning (PGD) Family closure.
4	14 female C.	Seizure onset 5 mo: GTC, myoclonic, tonic EEG: GEA Profound DD, progressive postnatal microcephaly, mild cerebral atrophy.	Trio ES	P Yes (PMID 25411445)	NM_016373.3 (*WWOX*): c.[140C>G]; [140C>G]	Reproductive planning (for siblings) Family closure. End diagnostic odyssey.
5	2 female NC.	Neonatal seizure onset to intractable MF. EEG: MFE + AB. Profound DD. Profound hypotonia evolving to spastic quadriplegia; cortical inattention.	Trio ES	LP No	NM_172107.2 (*KCNQ2*): c.[926C>T];[=]	Family closure.
6	13 female NC.	Seizure onset 6 mo: IS to tonic and focal EEG: hyps. Profound DD, hyperkinesis.	Trio ES	P No	NM_021007.2 (*SCN2A*): c.[680C>T];[=]	Family closure. End diagnostic odyssey.
7	4 female NC.	Neonatal onset seizures: FD, tonic. EEG: MEA Moderate DD, hypotonia to hypertonia.	Trio ES	P Yes (ClinVar)	NM_172107.2 *(KCNQ2*): c.740C>T; [=]	Choice of AED therapy. Family closure. Family support group access.
8	4 female NC (deceased).	Neonatal onset seizures. Tonic. EEG: normal to MEA Severe DD and ASD.	Trio ES	LP No	NM_021007.2 (*SCN2A*): c.[785T>C]	Family closure. End diagnostic odyssey. Guidance on AED (favoring Na channel blockers) Guidance on health surveillance (counseling on increased risk SUDEP).
9	1 male NC.	Seizure onset 5 mo: IS to drop, atypical absences, tonic and nonconvulsive status. EEG modified hyps. ‐to GEA Profound DD, hypotonia and hyperkinesis.	Trio ES	LP Yes (PMID: 25262651)	NM_004408.2 (*DNM1*): c.[709C>T];[=]	Family closure. Reproductive planning. Support groups. Research cohort. End diagnostic odyssey.
10	4 male NC.	Seizure onset 3 mo: focal tonic/opisthotonic posturing. IS (6 mo) to multiple types. EEG: MEA +AB Severe DD, ASD, focal pachygyria.	Trio ES	LP Yes (PMID: 23603762)	NM_001376.4 (*DYNC1H1*): c.[5884C>T];[=]	Family information. Family closure. End diagnostic odyssey.
11	5 male NC.	Seizure onset 8 mo: myoclonic and focal EEG: MEA Severe DD, stereotypies, self‐injurious behaviors, ataxia.	Trio ES	P Yes	NM_001008537.2 (*KIAA2022*): c.[1837G>T];[=]	Family closure (parental guilt). Family planning. End diagnostic odyssey. Family information
12	3 male NC.	Antenatal fetal hiccoughs. Focal to IS (4 mo), tonic, myoclonic and atypical absences. EEG: MEA +AB; hyps. Profound DD, cortical atrophy, hypoplastic CC, hypotonia.	Trio ES	LP Yes (PMID: 29069600)	NM_001287819.1 (*KCNT2*): c.[720T>A];[=]	Family information. Reproductive planning. End diagnostic odyssey. Family closure.
13	2 female NC.	Seizure onset 4 mo: IS to multifocal seizures. EEG: modified hyps. to MEA +AB Severe DD, ASD.	Trio ES	P Yes (PMID: 23934111)	NM_001099922.2 (*ALG13*): c.[320A>G];[=]	End diagnostic odyssey.
14	1 female NC (deceased)	Antenatal fetal hiccoughs. Initial focal to IS (5 mo) to tonic + myoclonic. EEG: MEA + AB Profound DD, nystagmus.	Trio ES	P Yes (PMID: 23934111)	NM_001099922.2 (*ALG13*): c.[320A>G];[=]	Family closure. Reproductive planning.
15	1 male NC	Seizure onset 6 weeks: GTC and tonic EEG: normal to bitemporal EA Severe DD, hypotonia, strabismus.	ST	LP No	NM_003159.2 (*CDKL5*): c.2420_2430delCCATTCATTCT	Family closure (relieved parental guilt). End diagnostic odyssey. Targeted management. Parental information.
16	2 male NC.	Seizure onset 11 mo: IS EEG: hyps. Moderate DD.	ST	P Yes (PMID: 16650978)	NM_139058.2 (*ARX*): c.[428_451dup]	Reproductive planning (for immediate and extended family). End diagnostic odyssey. Family closure. Family information.

AA, amino acid; AB, abnormal background; ACMG, American College of Medical Geneticists; AED, antiepileptic drug; ASD, autism spectrum disorder; B, benign; C, parental consanguinity; CC, corpus callosum; CP, cerebral palsy; D, damaging; DD, developmental delay; EA, epileptiform activity; EEG, electroencephalogram; ES, exome sequencing; FD, focal dyscognitive; GEA, generalized epileptiform activity; GTC, generalized tonic clonic; hyps., hypsarrhythmia; ID, intellectual disability; IS, infantile spasms; LP, likely pathogenic; MF, multifocal; MFE, multifocal epileptiform activity; mo, months; MRI, magnetic resonance imaging; NA, not applicable; NC, nonconsanguinity; P, pathogenic; PD, probably damaging; PGD, preimplantation genetic diagnosis; SUDEP, sudden unexpected death in epilepsy; ST, standard testing; T, tolerated.

Thirty‐two patients included in the study all underwent first‐tier testing and then standard second‐tier diagnostic testing including detailed metabolic studies, further diagnostic neuroimaging and single‐gene testing as determined by the involved clinicians (details and costs of first‐ and second‐tier tests provided in Table S1). Two of the 32 patients (individuals 15 and 16 in Table 1) received a diagnosis after standard second‐tier assessment, and these individuals did not proceed to exome sequencing. The remaining 30 individuals were enrolled in trio ES. Trio ES (proband and both parents) was conducted at the South East Area Laboratory Services (SEALS) Genetics laboratory and the Kinghorn Centre for Clinical Genomics (KCCG) as previously described (Palmer et al., 2015). Informed consent for ES was obtained.

**Figure 2:**
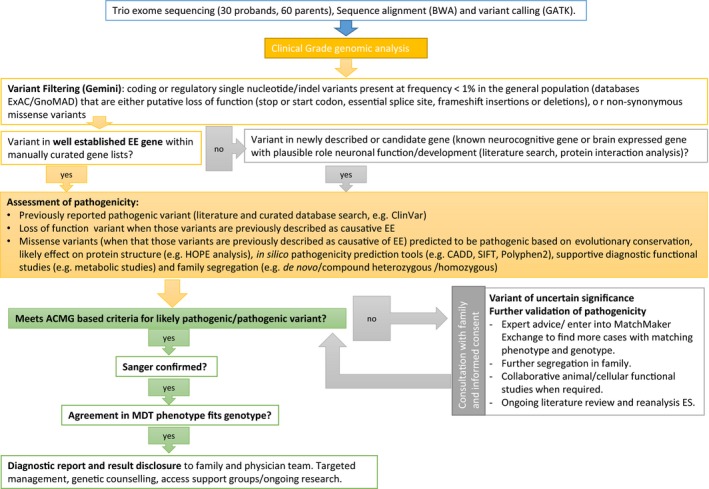
Flowchart of exome sequencing analysis. ACMG, American College of Medical Genetics; BWA, Burrows–Wheeler Alignment; EE, epileptic encephalopathy; ExAC, exome aggregation consortium; GATK, genome analysis toolkit; GnomAD, genome aggregation database; indel, insertion deletion; MDT, multidisciplinary team meeting

Candidate variants identified on trio ES were confirmed using Sanger sequencing. Assessment of the potential pathogenicity of candidate variants followed international guidelines (MacArthur et al., 2014; Richards et al., 2015). This included, when appropriate, a combination of segregation studies in the extended family, diagnostic functional studies, and in some circumstances, research functional studies (molecular biology, cell culture, and animal studies). Details of evidence for each pathogenic/likely pathogenic variant are in Table S2 and approach to variant classification provided in Figure 2. A diagnostic report was issued for pathogenic and likely pathogenic findings that were returned to the family by the involved clinical genetics and neurology physicians.

### 2.3 Diagnosis‐related cost ascertainment

The costs of all diagnostic‐related test and consultations were collected for each of the 32 patients entering the study (all tests and costs detailed Table S1). Case files, pathology databases, public hospital, and commercial costs were interrogated to collect diagnostic costs for each proband, and this information was entered in a deidentified database (ethics approval LNR/13/SCHN/112). Costs included the billed cost of the test, courier costs, work‐force costs associated with diagnostic procedures, patient admission for diagnostic tests such as imaging, and the fractional cost of specialist consultations related to diagnosis. We included costs related to diagnostically available functional tests that assisted in the assessment of pathogenicity of novel findings, for example, the cost of a repeat purine and pyrimidine screen to confirm a diagnosis of *ADSL‐*related disorder (MIM 103050) in patient 1. Functional validation of novel variants conducted in the collaborative research arena that did not entail direct institution or patient‐related costs were not included. Health costs collected for the trio ES pathway included DNA extraction, and all costs related to sequencing, the work costs of a Medical Genomicist to prioritize variants and of a genetic pathologist to assess pathogenicity and compile a diagnostic grade report.

### 2.4 Cost‐effectiveness analysis

In order to analyze the cost effectiveness of an exome diagnostic model (EDM) over a standard diagnostic model (SDM) we compared diagnostic yields and cost of diagnosis between the two models using a counterfactual approach similar to that described in Stark et al. (2017). This counterfactual approach allows comparison of diagnostic yields and costs under the SDM arm (i.e., first‐tier assessment and then standard second‐tier assessment) and the *actual/expected* diagnostic yields and costs under the EDM (i.e., first‐tier assessment and then second‐tier assessment including trio ES, Sanger confirmation of pathogenic/likely pathogenic variants, and adjunctive tests required to screen for causes of EE not readily diagnosable by ES: see Figure 1) *for the same individual*. Therefore, for this approach, the two patients who received a diagnosis in the SDM arm (individuals 15 and 16 in Table 1) were included as having received a diagnosis under the EDM, which was a reasonable assumptions as the *CDKL5* variant detected in patient 15 in the SDM was in a region adequately covered by the ES platforms used, and that screening for the common expansions in exon 2 of *ARX* is included in the EDM arm, which would have allowed detection of the *ARX* expansion variant present in patient 16 (Table 1). Therefore, for the health economic analysis *both* groups have a denominator of 32, although only 30 patients needed to proceed to ES.

We compared the average cost per patient and the average cost per diagnosis, between the SDM and the EDM. Both first‐ and second‐tier costs were included in both models. We also estimated an incremental cost per additional diagnosis (Drummond, Sculpher, Claxton, Stoddart, & Torrance, 2005) for the trio ES in the second‐tier testing pathway over the standard second‐tier testing pathway. We used bootstrap methods to estimate the uncertainty associated with outcomes. Bootstrapping is a widely used method to derive confidence intervals for outcomes (such as the incremental cost per additional diagnosis) for which the underlying distributions are not clear. Rather than making assumptions about the underlying distribution, bootstrapping relies on repeated samples drawn with replacement from the original data to create a sampling distribution (Drummond et al., 2005). Using this method, we created 1,000 replicated datasets and estimated outcomes such as average cost per patient, average cost per diagnosis, and incremental cost per additional diagnosis for each of the 1,000 replicated datasets. This generated 1,000 estimates of each outcome, which provided the sampling distribution of each outcome. Based on these sampling distributions, we estimated 95% confidence intervals for the outcomes using the percentile method (Briggs, Wonderling, & Mooney, 1997). Results were presented as scatter plots on a cost‐effectiveness plane. All analyses were performed in Microsoft Excel except for bootstrap simulations and confidence interval estimation, which were undertaken in SAS (SAS Institute, Cary, NC, USA) version 9.4.

### 2.5 Sensitivity analysis

As a sensitivity analysis, we substituted the price of trio ES provided by the in‐house SEALS service by the price provided by four different commercial laboratories: GeneDx, Maryland, United States; Fulgent Diagnostics, California, USA; Genome Diagnostics Nijmegen, Nijmegen, The Netherlands; and Centogene, Rostock, Germany, converted to Australian dollars (AU$) based on the exchange rate published by the Reserve Bank of Australia for 1 July 2016 (http://www.rba.gov.au/statistics/tables/xls-hist/2014-current.xls). Costs of these platforms are detailed in Table S1. To allow cost‐effectiveness analysis, an assumption was made that the diagnostic yield would be equivalent for all platforms, although it is appreciated that diagnostic yields may differ from company to company due to differences in, for example, sequencing bioinformatic methodology and variations in variant analysis and interpretation (Berg et al., 2017).

Costs were also assessed for an alternative high‐throughput sequencing (HTS) pathway where a commercial HTS EE panel was used instead of trio ES: the Courtagen EpiSeek Comprehensive (471 epilepsy‐related genes: Courtagen Life Sciences Inc., Massachusetts, USA) and the Fulgent Diagnostics EE panel (133 EE‐related genes). Costs of these panels as of 1 July 2016, converted to AU$ as above, are detailed in Table S1. To allow cost‐effectiveness analysis, the anticipated diagnostic yield for each panel was derived, using the assumption that if the gene was included in the HTS panel, the pathogenic variant detected in our cohort would be detected and therefore a diagnosis would be made for that patient.

## 3 RESULTS

Results were considered primarily in terms of comparison of diagnostic yield and cost effectiveness of the two diagnostic models.

### 3.1 Diagnostic yield

The diagnostic yield for the standard diagnostic arm was 2/32 (6.2%) compared to 16/32 (50%) for the in‐house “trio ES” arm (variants detected listed in Table 1 additional detail in Table S3), which, as discussed above, is under the assumption that both of the genetic diagnoses made in the SDM arm would have also been made in the EDM arm. Ten (56%) of 18 pathogenic/likely pathogenic variants (including compound heterozygous variants in two individuals) were novel. The clinical utility of each finding is shown in Table 1. Anticipated diagnostic yields for our cohort for the two commercial EE panels included were 9/32 (28%) for the Courtagen panel and 12/32 (37.5%) for the Fulgent panel.

### 3.2 Health costs of standard versus trio ES pathway

**Figure 3:**
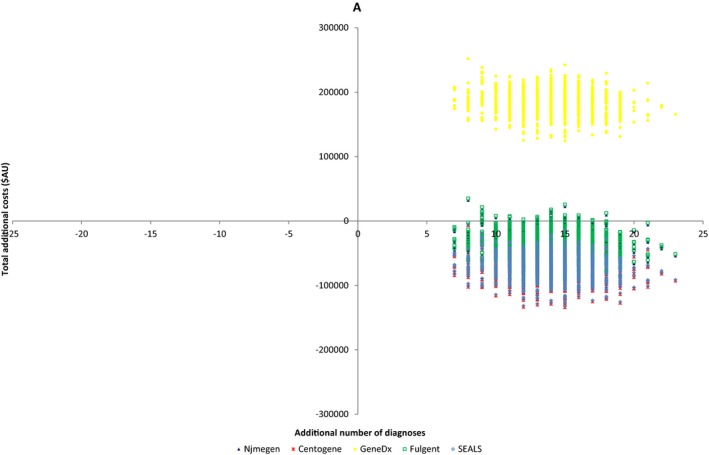
Cost‐effectiveness plane comparing “in‐house” (SEALS) and four commercial trio exome sequencing (ES) platforms (Centogene, Nijmegen, GeneDx, and Fulgent) to standard diagnostic pathway

The average total cost of diagnosis (first‐ and second‐tier tests) *per patient* for the standard pathway was AU$11,828 (95% CI: $10,677; $13,027) compared to AU$9,537 (95% CI: $9,412; $9,684) for the in‐house ES pathway. Given the improved diagnostic yield of the ES pathway (50% compared with 6.3%), this meant that the average cost of testing *per diagnosis* for the standard pathway was AU$189,243 (95% CI: $72,703; $406,142) compared to AU$19,074 (95% CI: $14,421; $27,969) for the ES pathway (i.e., the standard pathway cost about 10 times more per diagnosis). The ES pathway proved to be cost‐saving compared to the standard pathway, with a cost saving of AU$5,236 (95% CI: $2,483; $9,784) per additional diagnosis. The cost‐effectiveness plane (Figure 3) indicates that the trio ES diagnostic pathway is dominant (i.e., cost saving with a higher diagnostic rate), compared to the standard diagnostic pathway for all 1,000 bootstrapped replicated datasets.

### 3.3 Health costs for different commercial exome and high‐throughput sequencing panels

Anticipated costs of substituting our in‐house platform with four commercial trio ES platforms (Table 2) and the cost‐effectiveness plane are provided (Figure 3). The ES pathway was dominant (additional diagnoses while being cost saving) compared to the standard pathway for 100% of bootstrapped replicated datasets utilizing the Centogene ES platform, 97.2% of replicated datasets utilizing the Nijmegen ES platform and 96.1% of the replicated datasets using the ES Fulgent platform. GeneDx was the most expensive commercial platform analyzed, with both an increased diagnostic yield and an increased cost per patient compared to standard testing; translating to an increased cost of $13,113 (95% CI $8,610; $23,728) per additional diagnosis.

**Table 2:** Comparison of health costs (AU$) between standard diagnostic pathway and high‐throughput sequencing (HTS) pathways including “in‐house” and four commercial trio exome services and two commercial HTS panels (95% confidence intervals)

	Standard pathway	SEALS trio ES	Nijmegen trio ES	Centogene trio ES	GeneDx trio ES	Fulgent trio ES	Panel (Episeek comprehensive)	Fulgent EE panel
Cost per patient	11,827 (10,677; 13,027)	9,536 (9,412; 9,683)	10,698 (10,574; 10,845)	9,480 (9,356; 9,627)	17,564 (17,440; 17,711)	10,782 (10,658; 10,929)	10,179 (10,054; 10,326)	6,706 (6,582; 6,853)
Cost per diagnosis	189,242 (72,703; 406,141)	19,073 (14,421; 27,968)	21,397 (16,191; 31,348)	18,961 (14,335; 27,805)	35,129 (26,653; 51,322)	21,565 (16,319; 31,592)	36,193 (23,211; 81,817)	17,884 (12,183; 35,700)
Incremental cost per additional diagnosis compared to standard pathway		−5,236 (−9,784; −2,482)	−2,580 (−6,015; 100)	−5,364 (−9,987; −2,602)	13,113 (8,610; 23,728)	−2,388 (−5,777; 300)	−7,535 (−22,187; −2,277)	−16,386 (−35,120; −9,782)

EE, epileptic encephalopathy; ES, exome sequencing.

**Figure 4:**
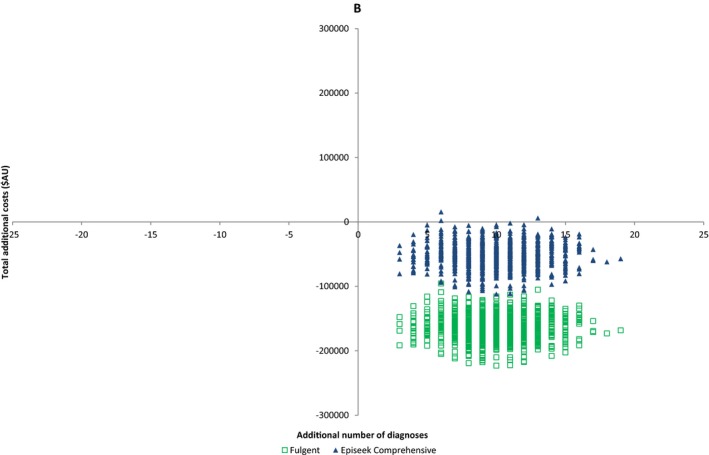
Cost‐effectiveness plane comparing two commercial high‐throughput sequencing (HTS) multigene platforms (Courtagen and Fulgent) to standard diagnostic pathway

Costs anticipated when substituting two commercial HTS EE panels into the counterfactual arm are provided (Table 2) and the cost‐effectiveness plane is shown (Figure 4). Both commercial HTS EE panels were an acceptable alternative to the standard pathway in terms of cost effectiveness, with more diagnoses than the SDM and less cost.

## 4 DISCUSSION

This study uniquely compares diagnostic yield, cost effectiveness, and clinical utility of the exome diagnostic model (EDM) over the standard diagnostic model (SDM) for patients with EE, who remain undiagnosed after first‐tier assessment. Our study focused on this group as they have traditionally entered an unsatisfactory, prolonged, costly, and invasive often unsuccessful “diagnostic odyssey.” We identified a need to assess the optimal pathway in the HTS era, to inform clinical practice and guide funding policies. This study reflects the type of patient cohort identified by Doble et al. (2016) as most likely to benefit from the early introduction of genomic testing to clinical medicine.

**Figure 5:**
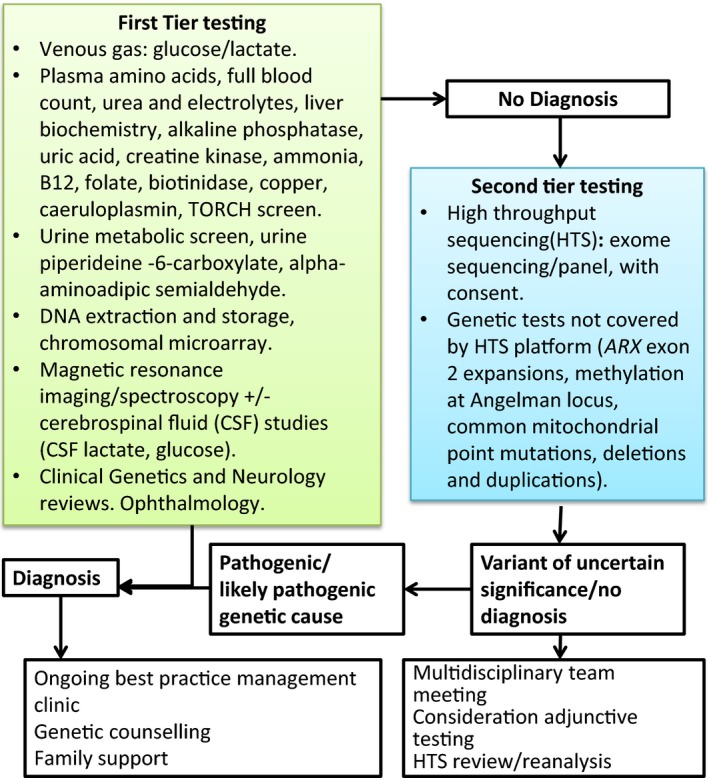
Recommended diagnostic pathway for epileptic encephalopathy incorporating high‐throughput sequencing

Our results strongly support the EDM, which resulted in a cost saving of AU$5,236 per additional diagnosis: approximately 10 times less expensive per diagnosis than the SDM. Sensitivity analysis demonstrated that the majority of commercial ES platforms analyzed were also cost‐effective, highlighting the robustness of our findings. We thus propose an optimal approach (Figure 5) for the investigation of EE which benefits patients and their families by reducing the length and invasiveness of the “diagnostic odyssey” and provides a cost saving for the health budget.

The diagnostic yield of the EDM was 50% compared to 6% for the SDM. This diagnostic yield is consistent with current literature (Helbig et al., 2016; Mercimek‐Mahmutoglu et al., 2015) and reflects the large number of genetic causes of EE (McTague et al., 2016), which the SDM is ill‐equipped to interrogate. We found, as others have, that a trio, as opposed to singleton, ES approach resulted in a high diagnostic yield: especially by facilitating detection of autosomal recessive and novel variants (Lee et al., 2014; Monroe et al., 2016). Our study also replicates other studies (Tarailo‐Graovac et al., 2016) that point to limitations of biochemical screening for diagnosis of neurometabolic causes of EE. Four patients received a diagnosis of a cryptic metabolic condition despite prior nondiagnostic metabolic screening (patients 1, 2, 13, 14 in Table 1). Although the majority of identified causal genetic variants in EE are autosomal and de novo (McTague et al., 2016), autosomal recessive causes are increasingly recognized (Helbig et al., 2016), as is germline mosaicism for dominant variants (Xu et al., 2015). In our cohort, five (31.3%) of the 16 diagnosed patients had an inherited X‐linked or autosomal recessive cause. This has critical genetic counseling implications.

The traditional “diagnostic odyssey” for EE patients can take years to complete (Kamien et al., 2012; Mercimek‐Mahmutoglu et al., 2015). By comparison, the quoted turnaround time for a commercial diagnostic trio ES is 6–12 weeks (source GeneTests: http://www.genetests.org, accessed June 2017). Therefore, ES can dramatically shorten the time to diagnosis providing earlier potential for targeted treatment/surveillance (Consortium E, 2015; Reif et al., 2017). Additional diagnoses will allow an increased number of families to access specific support groups and accurate genetic counseling, allowing options for antenatal and preimplantation genetic diagnosis (Berkovic, 2015). In our cohort, a diagnosis allowed several changes in management, including changes in choice and weaning of antiepileptic treatment, helped guide family planning, and in some circumstances helped families to move from a diagnostic to a palliative approach in the best interest of their children (Table 1). Even for those who do not reach a diagnosis through ES, there may be a deep satisfaction that “all that is possible” is being done to find the cause of EE (McGowan et al., 2013).

Although the cost of ES is falling, limited diagnostic budgets and concerns regarding the chance of detecting an incidental finding (IF) may mean that families and clinicians prefer to order a HTS gene panel (Chambers et al., 2016) over ES. Simpler genetic counseling (with a time and cost saving) is required for a panel than ES (Chambers et al., 2016). We therefore compared the cost and anticipated diagnostic yield of two commercial HTS EE panels with the SDM. Both assessed HTS panels were cost‐effective compared to the standard diagnostic model with a cost saving of AU$7,535 (95% CI: $2,278; $22,188) and AU$16,387 (95% CI: $9,782; $35,120) per additional diagnosis (Table 2 and Figure 3). The anticipated diagnostic yields reflect recent similar reported results (Mercimek‐Mahmutoglu et al., 2015; Trump et al., 2016), and demonstrated that a larger number of genes in a panel did not equate to a higher diagnostic yield, reflecting the importance of a tightly curated gene list (Chambers et al., 2016). For example, not all panels included the recently described EE genes *DNM1* (616346), *DYNC1H1* (614563), and *WWOX* (616211). This comparative analysis shows that trio ES leads to a greater diagnostic rate over multigene panel as this is an unbiased genome‐wide test which allows detection of novel and very recently described genetic causes of EE (Dyment et al., 2015; Helbig et al., 2016). No incidental findings (IF) were detected in the course of our study, unsurprisingly given the size of our cohort (Amendola et al., 2015; Olfson et al., 2015). IF can also result from HTS panels, for example, due to the overlap between cardiac arrhythmia and epilepsy genes (Chambers et al., 2016).

A major consideration in the genomic era is the rigor and costs required to establish pathogenicity of novel variants or genes in order to provide a secure diagnosis (Lee et al., 2014; McGowan et al., 2013; Monroe et al., 2016; Tarailo‐Graovac et al., 2016; Xu et al., 2015). The cost of variant analysis was therefore a major component of the cost of ES in our laboratory (Table S1) and the commercial HTS platforms that were analyzed as part of our sensitivity analysis. We utilized a conservative diagnostic‐grade approach to variant pathogenicity assessment, as recommended by international guidelines (Cartwright, 1997; Joshi et al., 2016). This entailed diagnostic functional assays, in silico prediction tools, literature review, interrogation of genomic databases of healthy and affected individuals (such as ExAC and ClinVar), and when required, collaborative clinical and basic science research (Briggs et al., 1997; Trump et al., 2016). It is envisaged that the costs related to variant analysis are likely to reduce in the future, as improved collaborative research and expanded genomic databases provide accurate pathogenicity assessments on more variants (Cartwright, 1997).

In considering our study design, we identified a need to assess the optimal diagnostic pathway in the genomic sequencing age, in order to inform best clinical practice and guide health funding policy. Although the temptation to opt for ES as a first‐tier genetic test is strong, and in some circumstances may be valid (Whole‐exome sequencing strategy proposed as first‐line test, 2016), it is clear that with the complexities associated with genetic testing, phenotypic/genotypic heterogeneity and unique nuances of specific clinical situations, a well‐thought‐out approach is required to result in optimal outcomes, in terms of patient care and cost effectiveness. To this end, we have proposed a diagnostic pathway (Figure 5) derived from the EDM used in this study. We retain a first‐tier diagnostic step to allow for timely screening for rapidly diagnosable conditions and disorders which would require an immediate change in patient management. Multidisciplinary assessment of neuroradiology in this first tier is increasingly recognized to be critical in assessing for subtle cortical malformations that are less likely to have a germline genetic cause (Berg et al., 2017), and we appreciate that the access of all patients to such tertiary neuroradiology review is not available in all centers, which could lead to a reduction in diagnostic yield of second‐tier testing. The second‐tier diagnostic step integrates ES or multigene panel, as well as those additional tests required for a comprehensive second‐tier evaluation (e.g., screening for methylation abnormalities, triplet repeat and non‐nuclear mitochondrial disorders) and multidisciplinary team (MDT) meetings. We also recommend pre‐ and posttest counseling and reanalysis of the HTS data for undiagnosed patients, as this has been shown to lead to further diagnoses over time (Stark et al., 2016; Wenger et al., 2016).

We have demonstrated that a two‐tier diagnostic approach, incorporating trio ES as a second‐tier test, offered a significantly higher diagnostic yield and improved cost‐effectiveness compared to a standard diagnostic approach for EE. We were able to show reproducibility of our findings by analyzing costs and anticipated diagnostic yields associated with commercial ES platforms and multigene panels. We therefore propose that a high‐throughput sequencing approach, incorporating trio ES or gene panel, depending on resources and patient and clinician preference, would be an appropriate second‐tier diagnostic step for patients with EE who remain undiagnosed after first‐tier testing (Figure 5), with implications for clinical practice and health policy (Monroe et al., 2016; Soden et al., 2014).

## ACKNOWLEDGMENTS

The authors thank patients’ families for their participation in this study; the clinical neurology, general pediatric, and clinical genetics teams involved with the clinical care and support of the patients; and the expert technical assistance of Toni Saville, Glenda Mullan, and Corrina Walsh from SEALS Genetics Laboratory and from Garvan Institute. This work was supported by the Kinghorn Foundation and the National Health and Medical Research Council (GNT11149630 to E.P., GNT0512123 to T.R.).

## 5 CONFLICT OF INTEREST

None declared.
