# Supplementary material for: Integrating exome sequencing into a diagnostic pathway for epileptic encephalopathy: Evidence of clinical utility and cost effectiveness
Source: Mol Genet Genomic Med. 2018 Jan 4;6(2):186–99. doi: 10.1002/mgg3.355 (PMC5902395; doi:10.1002/mgg3.355)
Supplement: Supplementary file 1 [file MGG3-6-186-s001.docx]

# SUPPLEMENTARY DATA

**Supplementary Table 1: Details of investigations and associated costs included in first and second-tier testing**

| Test | Laboratory | Test cost (AU$) | First-tier (FT) or second-tier (ST) |
| --- | --- | --- | --- |
| Biochemistry, haematology, metabolic and infection screen | | | |
| Basic biochemistry: urea, electrolytes, lactate, ammonia, liver biochemistry, alkaline phosphatase, uric acid, creatine kinase | SEALS, Sydney | 17.70 | FT |
| Venous blood gas | SEALS, Sydney | 42.60 | FT |
| Full blood count | SEALS, Sydney | 16.95 | FT |
| TORCH screen (Toxoplasma, Rubella, Syphilis, Cytomegalovirus and HSV congenital infection screen) | SEALS, Sydney | 69.10 | FT |
| Urine metabolic screen | CHW, Sydney | 200.00 | FT |
| B12, folate | SEALS, Sydney | 42.95 | FT |
| Copper, ceruloplasmin, selenium, zinc | SEALS, Sydney | 52.45 | FT |
| Plasma amino acids | CHW, Sydney | 100.00 | FT |
| AASA and P6C | VCGS, Melbourne | 150.00 | FT |
| Iron studies | SEALS, Sydney | 32.55 | ST |
| Vitamin D | SEALS, Sydney | 39.05 | ST |
| Thyroid function including rT3/T4 | SEALS, Sydney | 83.35 | ST |
| 7 dehydrocholesterol | CHW, Sydney | 175.00 | ST |
| Total homocysteine | CHW, Sydney | 100.00 | ST |
| White cell enzymology (lysosomal enzymes) | SA Pathology, Adelaide | 390.00 | ST |
| Transferrin isoforms | CHW, Sydney | 210.00 | ST |
| O-glycans | Mater Pathology, Brisbane | 33.10 | ST |
| Acyl carnitine profile | CHW, Sydney | 200.00 | ST |
| Buffy coat electron microscopy | Concord, Sydney | 200.00 | ST |
| Very long chain fatty acids | CHW, Sydney | 175.00 | ST |
| Biotinidase | CHW, Sydney | 120.00 | ST |
| Purines and pyrimidines | VCGS, Melbourne | 300.00 | ST |
| Guanadino compounds | CHW, Sydney | 225.00 | ST |
| Urinary pterins | CHW, Sydney | 39.00 | ST |
| Urinary oligosaccarides | SA Pathology, Adelaide | 145.00 | ST |
| CSF: blood lactate; CSF: blood glucose | SEALS, Sydney | 17.70 | FT |
| CSF neurotransmitters | CHW, Sydney | 39.00 | ST |
| CSF amino acids | CHW, Sydney | 100.00 | ST |
| CSF folate | CHW, Sydney | 39.00 | ST |
| Respiratory chain enzymology: muscle and liver | MCRI, Melbourne | 1,130.00 | ST |
| Respiratory chain enzymology: fibroblasts | MCRI, Melbourne | 1,525.00 | ST |
| Genetic testing | | | |
| DNA extraction and storage | SEALS, Sydney | 250.00 | FT |
| Chromosomal microarray proband | SEALS, Sydney | 589.90 | FT |
| Chromosomal microarray - parent one | SEALS, Sydney | 589.90 | FT |
| Chromosomal microarray - parent two | SEALS, Sydney | 589.90 | FT |
| Screening for common expansions in *ARX* | SA Pathology, Adelaide | 340.00 | ST |
| Common mitochondrial point mutations | VCGS, Melbourne | 350.00 | ST |
| Common mitochondrial deletions and duplications | VCGS, Melbourne | 400.00 | ST |
| Methylation at Angelman locus | CHW, Sydney | 300.00 | ST |
| Screening for expansions in *FMR1* | SEALS, Sydney | 101.30 | ST |
| Sequencing of *UB3EA* | Mater Pathology, Brisbane | 650.00 | ST |
| Sequencing of *CDKL5* | CHW, Sydney | 1,000.00 | ST |
| Sequencing of *MECP2* | CHW, Sydney | 600.00 | ST |
| Sequencing of *SCN1A* | GTG, Melbourne | 1,999.00 | ST |
| Sequencing of *STXBP1* | Lab plus, Auckland, New Zealand. | 1,339.70 | ST |
| Sequencing of *GLDC* | Lab plus, Auckland, New Zealand. | 1,982.78 | ST |
| Cost of trio exome sequencing | SEALS, Sydney | 4,036.55 | ST |
| Cost of sanger sequencing segregation in trio | SEALS, Sydney | 750.00 | ST |
| Cost of trio exome sequencing | Nijmegen | 5,350.93 | ST |
| Cost of trio exome sequencing | Centogene | 4,094.57 | ST |
| Cost of trio exome sequencing | GeneDx | 12,361.70 | ST |
| Cost of EE NGS panel | Fulgent | 1,183.77 | ST |
| Cost of EE NGS panel | CHW, Sydney | 1,500.00 | ST |
| Cost of EE NGS panel | Courtagen-focus | 2,769.85 | ST |
| Cost of EE NGS panel | Courtagen-extended | 1,217.96 | ST |
| Cost of EE NGS panel | Centogene | 5,279.97 | ST |
| EEG/neuroimaging | | | |
| EEG | | 123.10 | FT |
| Neuroimaging procedure related costs | | 1,264.48 | FT/ST |
| MRI head scan | | 403.20 | FT/ST |
| PET head scan | | 918.00 | ST |
| Lumbar puncture | | 75.30 | FT |
| MRS head scan | | 336.00 | ST |
| Tc99m radionucleotide scan | | 348.10 | ST |
| CT head scan | | 250.00 | ST |
| Workforce costings | | | |
| Specialist hospital clinic appointment (MBS item 132) | | 263.90 | |
| Specialist hospital clinic (MBS item 133) | | 132.10 | |
| Specialist hospital clinic (MBS item 131) | | 79.75 | |
| Specialist case management (Senior staff specialist level/hour) | | 142.00 | |
| Genetic counsellor case management (Associate genetic counsellor/hour) | | 33.60 | |
| Specialist trainee case management (fellow/hour) | | 52.80 | |
| Specialist trainee case management (year 2 registrar/hour) | | 48.10 | |
| Inpatient specialist consultation | | 150.90 | |
| Specimen courier costs | | | |
| Across Sydney (same day) for metabolic testing | | 48.50 | |
| Sydney to Melbourne | | 11.66 | |
| Sydney to Adelaide | | 15.00 | |
| Sydney to Brisbane | | 11.66 | |
| Sydney to Auckland, New Zealand | | 36.11 | |

**Abbreviations**

AASA: alpha-aminoadipic semialdehyde; CSF: cerebrospinal fluid; CT: Computed tomography; EE: epileptic encephalopathy; EEG: electroencephalogram; FT: first-tier: MBS: Medicare benefit scheme; MRI: magnetic resonance imaging**;** MRS: magnetic resonance spectroscopy; NGS: next generation sequencing;P6C: piperideine-6-carboxylate; PET: positron emission tomographyST: second-tier.

**Supplementary Table 2: Demographic details of cohort**

|  | **Total cohort n=32; (% of total cohort)** | **Diagnosed group; n=16 (% of diagnosed)** | **Undiagnosed group n=16 (% of undiagnosed)** |
| --- | --- | --- | --- |
| **Age** |  |  |  |
| 0-2 | 5 (16%) | 4 (25%) | 1 (6.5%) |
| 2-5 | 14 (43%) | 7 (43.5%) | 7 (43.5%) |
| 5-10 | 8 (25%) | 1 (6.5%) | 7 (43.5%) |
| 10-15 | 5 (16%) | 4 (25%) | 1 (6.5%) |
| **Gender** |  |  |  |
| Male | 17 (53%) | 7 (44%) | 10 (63%) |
| Female | 15 (47%) | 9 (56%) | 6 (37%) |
| **Parental consanguinity?** |  |  |  |
| Yes | 3 (9%) | 2 (12.5%) | 1 (6.5%) |
| No | 29 (91%) | 14 (87.5%) | 15 (93.5%) |
| **Subtype of EE** |  |  |  |
| Infantile Spasms | 15 (47%) | 8 (50%) | 7 (43.5%) |
| Neonatal onset seizures | 4 (13%) | 3 (18.5%) | 1 (6.5%) |
| Intractable infantile onset EE (not spasms) | 11 (34%) | 4 (25%) | 7 (43.5%) |
| ‘Evolving’ EE (EEG does not initially meet diagnostic criteria for EE, but evolves to do so) | 2 (6%) | 1 (6.5%) | 1 (6.5%) |

Abbreviations: EE epileptic encephalopathy; EEG electroencephalogram

**Supplementary Table 3: Phenotypic summary of affected individuals and details of pathogenic/likely pathogenic variants detected.**

| Family | Age (yr)  Gender  Parental  consanguinity? | Phenotype (seizure and EEG semiology; developmental and neurological features) | | Diagnosis? ( method) | Inheritance | ACMG  Classification  reported (PMID) | Variant (s)  GRCh37): | Evidence of Pathogenicity:  (*in silico* pathogenicity predictors;  MAF in v0.3 ExAC database;  protein domain affected;  functional validation? |
| --- | --- | --- | --- | --- | --- | --- | --- | --- |
| 1 | 11;  female;  NC. | Seizure onset 4 mo: IS responding to UKISS and persistent multifocal /tonic seizures.  EEG: multifocal epileptogenic activity (slow bilateral occipital/temporal spikes) and abnormal background)  Profound DD.  Dystonia. cortical inattention, slowly deteriorating course. | | Yes  Trio ES | AR compound het | LP; Yes (PMID: 10888601). | NM_000026.2 (*ADSL*):  c.[1288G>A];  [1370T>C] p.[(Asp430Asn)];  p.[(Val457Ala)]. | c.[1288G>A]:Polyphen2 PD ; SIFT D; CADD 23.8:  c. [1370T>C]: Polyphen2 PD ;SIFT D; CADD 23.8;  c.[1288G>A] MAF in ExAC: 1.647X10^-5^  c.[1370T>C] Not listed in ExAC;  adenylosuccinate lyase domain;  abnormal repeat purine and pyrimidine screen, |
| 2 | 11;  male;  NC | Seizure onset 4 mo: tonic and complex partial. Intractable tonic, tonic clonic and focal seizures.  EEG multifocal epileptogenic foci abnormal background.  Profound DD and developmental regression. Hypotonia, progressive postnatal microcephaly and cerebral atrophy on MRI. | | Yes  Trio ES | AR compound het | P; Yes (PMID: 26318253) | NM_183356.3  *(ASNS)*:  c.[866G>C];  [1010C>T] p.[(Gly289Alal)];  [(Thr337Ile)] | c.[866G>C]: Polyphen2 PD SIFT D; CADD 24.8:  c.[1010C>T]: Polyphen 2 B; SIFT D; CADD 20.9;  Not listed in OmIM;  altered residues near ATP binding pocket;  reduced proliferation of patient fibroblasts when cultured in asparagine limited growth conditions. |
| 3 | 1;  female;  C. | Seizure onset 4 mo: focal progressing to intractable multifocal seizures.  EEG: modified hypsarrhythmia, multifocal epileptogenic activity, abnormal background  Profound DD, progressive deterioration. Congenital retinopathy. Died at 12 mo. | | Yes  Trio ES | AR homozygous | LP; Yes (PMID 27270415) | NM_022786.1  *(ARV1):*  c.[294+1G>A]; c.[294+1G>A]  p.[Lys59_Asn98del];[Lys59_Asn98del]. | No *in silico* predictors (splice variant);  ExAC MAF 1.653X10^5^ (heterozygous only)  Variant shown to abolishe a splice site between exons 2 and 3 resulting in loss of gene expression and protein function in cell line and yeast model. |
| 4 | 14;  female;  C. | Seizure onset 5 mo: GTC, myoclonic, tonic seizures.  EEG: generalised epileptiform activity.  Profound DD. Progressive postnatal microcephaly.  Mild cerebral atrophy on MRI. | | Yes  Trio ES | AR homozygous | P; Yes (PMID 25411445) | NM_016373.3  *(WWOX)*:  c.[140C>G];[140C>G] p.[(Pro47Arg)]; [(Pro47Arg)] | Polyphen2 PD; SIFT D; CADD 23.3;  Not listed in ExAC;  substitution of hydrophobic aa in core of WW domain with hydrophilic amino acid. |
| 5 | 2;  female;  NC. | Neonatal seizure onset: progressing to intractable multifocal seizures.  EEG: multifocal epileptogenic activity; abnormal background.  Profound DD.  Profound hypotonia evolving to spastic quadriplegia; cortical inattention. Died aged 2. | | Yes  Trio ES | AD *de novo* | LP; No | NM_172107.2  (*KCNQ2)*:  c.[926C>T];[=] p.[(Ala309Val)];[=] | Polyphen2 PD; SIFT D; CADD 19;  Not listed in ExAC;  S6 transmenbrane segment of pore domain of potassium channel;  variants in same segment shown to markedly reduce KCNQ2 function^.^ |
| 6 | 13;  female;  NC. | Seizure onset 6 mo: IS. Evolved to intractable tonic and focal seizures.  EEG hypsarrythmia. Initial temperature sensitivity. Developmental stagnation with seizure onset and profound DD. Hyperkinetic arm movements. | | Yes  Trio ES | AD *de novo* | P; No | NM_021007.2  (*SCN2A)*: c.[680C>T];[=] p.[(Thr227Ile)];[=] | Polyphen2 PD; SIFT D; CADD 21;  Not listed in ExAC;  S4 transmenbrane segment of first pore domain of sodium channel;S4 harbours voltage sensor, which regulates channel activation. |
| 7 | 4;  female;  NC. | Neonatal onset seizures. Focal dyscognitive, tonic:seizures initially intractable seizure disorder, with improved control by 4 years.  EEG: multifocal epileptogenic activity  Moderate DD with developmental progress. Hypotonia evolving to hypertonia. | | Yes  Trio ES | AD *de novo* | P; Yes (ClinVar) | NM_172107.2  (*KCNQ2)*:  c.[740C>T]; [=]  p.[(Ser247Leu)];[=] | Polyphen2 B; SIFT D; CADD 25.9;  Not listed in ExAC;  S5 transmenbrane segment (pore domain of potassium channel). |
| 8 | 4;  female;  NC. | Neonatal onset seizures- tonic. Remains intractable.  EEG: ,normal evolving to multifocal epileptogenic activity;  Severe DD and ASD. | | Yes  Trio ES | AD *de novo* | LP;No | NM_021007.2  (*SCN2A)*:  c.[785T>C];[=] p.[(Phe262Ser)];[=] | Polyphen2 PD; SIFT D; CADD 25.6;  Not listed in ExAC;  S5 transmenbrane segment (first pore domain of sodium channel) where *de novo* variants missense variants previously reported in EE. |
| 9 | 1;  male;  NC. | Seizure onset 5 mo: IS evolving to intractable multi-daily seizures: drop, atypical absences, generalised tonic seizures and non-convulsive status.  EEG modified hypsarrhythymia to generalised slowing/ generalised epileptiform activity. Profound DD but developmental progress. Hypotonia and hyperkinetic. | | Yes  Trio ES | AD *de novo* | LP:Yes (PMID: 25262651) | NM_004408.2  (*DNM1*:  c.[709C>T];[=] p.[(Arg237Trp)];[=] | Polyphen2 PD; SIFT D; CADD34;  Not listed in ExAC  dynamin central/GTPase domain.. |
| 10 | 4;  male;  NC. | Seizure onset 3 mo: focal tonic/ opisthotonic posturing. .IS at 6 mo progressing to multiple seizure types.  EEG: multifocal epileptiform activity, asymmetric slow background and decrements.  Autistic features and severe DD, developmental progress.  MRI: Simplification gyri and focal pachygyria. | | Yes  Trio ES | AD *de novo* | LP; Yes (PMID: 23603762) | NM_001376.4  (*DYNC1H1)*:  c.[5884C>T];[=] p.[(Arg1962)];[=] | Polyphen2 PD; SIFT D; CADD 35;  Not listed in ExAC;  AAA1 (ATPase) domain, part of motor domain of protein; essential for protein motility. |
| 11 | 5;  male;  NC. | Seizure onset 8 mo: persistent myoclonic and focal seizures. Evolving EEG with multifocal epileptogenic activity.  Severe DD, developmental progress.  Stereotypies and self-injurious behaviours. Unsteady gait. | | Yes  Trio ES | AD *de novo* | P;Yes | NM_001008537.2  (*KIAA2022)*  :c.[1837G>T];[=] p.[(Glu613*)];[=] | Predicted to result in premature stop codon; the resulting transcript likely to be targeted for nonsense mediated decay;  Not listed in ExAC  LoF variants reported in multiple individuals with EE. |
| 12 | 3;  male;  NC. | Antenatal fetal hiccoughs. Initial focal seizures evolving to IS (4 mo). Intractable prolonged tonic; myoclonic jerks and atypical absences.  EEG disorganised background, decrements, multifocal epileptogenic activity/ hypsarrhythmia. Profound DD but developmental progress.  MRI: Cortical atrophy and thin corpus callosum, hypotonia. | | Yes  Trio ES | AD *de novo* | LP;Yes (ClinVar;http://dx.doi.org/10.1016/j.celrep.2017.09.088) | NM_001287819.1 *KCNT2*):  c.[720T>A];[=]  p. [(Phe240Leu)];[=] | Polyphen PD; SIFT T; CADD 25.5;  Not listed in ExAC  affected residue demonstrated critical for channel gating and functional studies in *Xenopus* oocytes demonstrate variants alters channel electrophysiological properties. |
| 13 | 2;  female;  NC. | Seizure onset 4 mo: IS. Progressed to intractable multifocal seizures.  EEG: modified hypsarrhythmia evolved to slowing and multifocal epileptiform discharges.  Severe DD but with some developmental progression. Autistic features. | | Yes  Trio ES | XL *de novo* | P; Yes (PMID: 23934111) | NM_001099922.2  (*ALG13)*:  c.[320A>G];[=] p.[(Asn107Ser)];[=] | Polyphen2 PD; SIFT D; CADD 23.7;  Not listed in ExAC |
| 14 | 1;  female;  NC. | Antenatal fetal hiccoughs. Initial focal seizures evolving to IS (5 mo). Persistent intractable seizures (tonic; myoclonic).  EEG multifocal and abnormal background. Profound DD with no developmental progress after seizure onset. Nystagmus. Died aged 1. | | Yes;  Trio ES | XL *de novo* | P; Yes (PMID: 23934111) | NM_001099922.2 *(ALG13)*:  c.[320A>G];[=];  p.[(Asn107Ser)];[=] | Polyphen2 PD; SIFT D; CADD 23.7;  Not listed in ExAC |
| 15 | 1;  male;  NC. | Seizure onset 6 weeks: tonic clonic and tonic  EEG initially normal progressing to abnormal with bitemporal epileptogenic activity.  Severe DD, hypotonia, strabismus. | | Yes;  ST | XL mat inherited (mosaic) | LP; No | NM_003159.2 *(CDKL5)* c.2420_2430delCCATTCATTCT p.[(Ser807fs*2)] | Predicted frameshift;  Not listed in ExAC  C-terminus of *CDKL5* regulates the kinase activity of the protein, sub-cellular localisation and function;  Truncating mutations from AA 805 described in EE. |
| 16 | 2;  male;  NC. | Seizure onset 11 mo: IS  EEG: hypsarrhythmia  Moderate developmental delay with developmental progress. | | Yes:  ST | XL mat inherited | P; Yes (PMID: 16650978) | NM_139058.2  *(ARX)*: c.[428_451dup] p.[(Ala150_Ala151insGlyAlaAlaAlaAlaAlaAlaAla)] | Well established recurrent expansion mutation causative of EE phenotype in males^;^  Not listed in ExAC |
| 17 | 7;  female;  NC. | Seizure onset 4 mo: intractable focal seizures.  EEG multifocal epileptogenic activity.  Profound ID. Scoliosis and dislocated hips, truncally hypotonic with increased appendicular tone. | | No | NA | NA | NA | NA |
| 18 | 6;  male;  NC. | Seizure onset 5 mo; focal dyscognitive to tonic, tonic clonic  EEG: slow waves, multifocal spikes, slow background  Severe to profound ID (step-wise deterioration); dyskinetic movement disorder, | | No | NA | NA | NA | NA |
| 19 | 6;  male;  NC. | Seizure onset 6 mo: IS evolving to tonic, GTC and focal.  EEG:modified hypsarrhythmia and multifocal epileptiform activity  Severe ID. Motor stereotypies and athetoid movements. MRI: cortical atrophy. | | No | NA | NA | NA | NA |
| 20 | 4;  male;  NC | Seizure onset 4 mo: IS progressing to atypical absences and focal seizures.  EEG: modified hypsarrythmia  Moderate ID | | No | NA | NA | NA | NA |
| 21 | 5;  male;  NC | Seizure onset 11 mo: atypical absences and drops  EEG: generalised epileptiform activity  Severe ID | | No | NA | NA | NA | NA |
| 22 | 3;  male;  NC. | Seizure onset 3 mo: atypical IS progressing to tonic and focal dyscognitive seizures.  EEG: modified hypsarrhythmia; progressing to multifocal, disorganised background  Mild ID and autistic features, hyperactivity | | No | NA | NA | NA | NA |
| 23 | 5;  female;  NC. | | Seizure onset 5 weeks: multifocal seizures.  EEG: hypsarrhythmia: evolving to multifocal epileptiform activity with abnormal background  Moderate ID. | No | NA | NA | NA | NA |
| 24 | 10;  female;  NC. | | Seizure onset 4 mo: IS progressing to generalised tonic, absence and atonic seizures.  EEG: multifocal epileptiform activity and abnormal background Severe ID, autistic features | No | NA | NA | NA | NA |
| 25 | 4;  male;  NC. | | Seizure onset 11 mo: tonic progressing to tonic clonic, atypical absence, startles.  EEG: mutifocal epileptiform activity and abnormal background  Moderate ID and autistic features. | No | NA | NA | NA | NA |
| 26 | 4,  male;  C. | | Seizure onset 10 mo: myoclonic jerks, GTC evolving to tonic and atypical absences  EEG: multifocal and generalised epileptiform activity with background slowing.  Profound ID, cortical visual impairment, hyperkinetic movement disorder, hypotonia. MRI cerebral atrophy. | No | NA | NA | NA | NA |
| 27 | 5;  male;  NC | | Seizure onset 6 mo: IS  EEG: multifocal epileptiform activity  Seizures controlled with VNS  Moderate ID | No | NA | NA | NA | NA |
| 28 | 3;  female;  NC | | Seizure onset neonatal: tonic  EEG :encephalopathic  No developmental progress: profound ID | No | NA | NA | NA | NA |
| 29 | 6;  male;  NC | | Seizure onset 5 mo: IS; tonic seizures  EEG: modified hypsarrhythmia evolving to multifocal epileptogenic activity with abnormal background  Severe ID, autistic features  Hypotonia, hyperactivity | No | NA | NA | NA | NA |
| 30 | 3;  female;  NC | | Seizure onset 4mo: focal dyscognitive, drops,  EEG: multifocal epileptiform activity.  Mild-moderate ID and autistic features | No | NA | NA | NA | NA |
| 31 | 4;  female;  NC | | Seizure onset 6mo: atypical IS  EEG: multifocal epileptiform activity and abnormal background  Mild to moderate ID and autism | No | NA | NA | NA | NA |
| 32 | 1;  male;  NC | | Seizure onset 3 mo: atypical asymmetric -IS  EEG multifocal epileptiform activity and abnormal background  MRI cortical atrophy  Moderate ID | No | NA | NA | NA | NA |

**Abbreviations:** AA amino acid; ASD autism spectrum disorder; B benign; C parental consanguinity; D damaging; DD developmental delay; EEG electroencephalogram; GTC generalised tonic clonic; ID intellectual disability; IS infantile spasms; LP likely pathogenic; Mo months; MRI magnetic resonance imaging; NA not applicable; NC non-consanguinity; P pathogenic; PD probably damaging; T tolerated; VNS vagus nerve stimulator
